# Technology readiness among UK-based cognitively unaffected older adults during the COVID-19 pandemic: potential implications for decentralised alzheimer’s disease prevention trials

**DOI:** 10.1186/s12877-025-06158-3

**Published:** 2025-07-30

**Authors:** S. Taylor, L. Dunn, C. Udeh-Momoh, K. Abbott, P. Giannakopoulou, L. Middleton, O. Robinson, J. Kalsi, D. Kafetsouli, J. Ford

**Affiliations:** 1 Ageing Epidemiology (AGE) Research Unit, School of Public Health, Imperial College London, London, UK; 2 School of Public Health Sciences, Wake Forest University School of Medicine, Winston-Salem, NC USA

**Keywords:** Alzheimer’s disease, Dementia, Technology, COVID-19, Older Adults, Telemedicine

## Abstract

**Background:**

Alzheimer’s disease (AD) remains a global health and socioeconomic burden. Telemedicine has been more widely used since the beginning of the COVID-19 pandemic and may be an effective strategy to mitigate the rising costs associated with AD. This study aimed to assess technology readiness among older adults at risk of developing dementia, with the goal of informing the design and delivery of technology-based approaches in AD prevention research.

**Methods:**

Cognitively unaffected older adults (*n* = 226) from the CHARIOT PRO Substudy were invited to complete the CHARIOT Technology Questionnaire (CTQ). CTQ assessed technology experiences and attitudes, including ‘technology readiness’ via the Technology Readiness Index (TRI).

**Results:**

Female participants scored, on average, lower TRI (M = 27.50, SD = 6.87) compared to males (M = 29.50, SD = 6.02). Furthermore, age predicted levels of technology readiness. Exploratory factor analysis determined two factors: “Technology Competence” (Factor 1) and “Technology Trepidation” (Factor 2). Gender differences were found for “Technology Competence” (but not “Technology Trepidation”), and age predicted “Technology Trepidation” (but not “Technology Competence”).

**Discussion:**

Differences in gender, age, “Technology Competence”, and “Technology Trepidation” may highlight those who need additional study and/or support in remote-based AD dementia prevention trials.

**Conclusions:**

COVID-19 has accelerated our adoption of ‘digitalisation’ in AD dementia research. A deeper understanding of the barriers to technology readiness may help inform future AD research studies.

**Trial registration:**

The CHARIOT PRO SubStudy is registered with clinicaltrials.gov (NCT02114372).

## Introduction

Alzheimer’s disease (AD) is the most common cause of dementia, accounting for 60 to 70% of worldwide dementia cases [1]. The prevalence of older adults with dementia in the United Kingdom (UK) was estimated at 885,000 in 2019, and this is projected to increase to 1.5 million by 2040 [2]. AD is a neurodegenerative disease characterised by progressive cognitive and behavioural impairment [3] and loss of social functioning and independence [4]. AD pathophysiological processes may occur decades before the detection of clinical symptoms [5, 6]. Therefore, early identification is critical to provide a greater opportunity for potential therapeutic intervention before significant cognitive impairment manifests [6]. Early diagnoses could also reduce the economic burden of AD by lessening associated care and hospitalisation requirements [7].

AD is a significant burden on the UK’s National Health Service (NHS), costing UK hospitals £2.70 billion in 2017/2018 [8]. The burden on UK health and social care systems is also expected to inflate as AD prevalence increases [2]. Costs associated with dementia were reported at £34.70 billion in 2019, projected to grow to £94.10 billion by 2040, with healthcare costs accounting for 14%, social care for 45%, and unpaid care for 40% in 2019 [2]. Although memory clinics pose a cost-effective strategy compared to primary care facilities, these services are often at capacity [9].

An alternative approach to alleviate financial and capacity burdens may be to implement remote assessments, such as for those with memory difficulties. “Digitalisation” affects all aspects of socio-cultural domains [10], and telemedicine has become a significant digitalisation feature within health and social care services, with increased adoption throughout the Coronavirus disease 2019 (COVID-19) pandemic [11, 12]. Although telemedicine challenges the traditional face-to-face service model, it poses an opportunity to address some healthcare system challenges by facilitating greater communication with healthcare providers and quicker access to care [12]. To reduce the spread of COVID-19 in Great Britain, public health safety measures were implemented (“*Coronavirus: Strict new curbs on life in UK announced by PM*”, [13]. Consequently, COVID-19 enabled a greater adoption of telemedicine models [14]. In AD research, clinical trials such as the TRAILBLAZER-ALZ 3 study have adopted decentralised approaches to enhance geographic reach and increase ethnic and racial diversity among participants [15]. This multicentre Phase 3 trial evaluated the effects of donanemab versus placebo in cognitively unimpaired older adults with biomarker evidence of AD pathology [15].

However, despite the technological advances within the medical field, pre-COVID, the neuropsychology field still relied predominantly on in-person neuropsychological assessments [16]. Yet, the evidence demonstrated that administering remote neuropsychological cognitive assessments with older adults, either cognitively unaffected or diagnosed with dementia, yielded reliable and valid results when compared to in-person testing [17, 18]. Furthermore, in research settings, remote neuropsychological assessment approaches could be a cost-effective method to support improving health outcomes in older adults. For example, AD studies that plan to implement remote protocols, such as, but not limited to, AD-Riddle, may aid in furthering our understanding of precision medicine [19].

AD prevention trials required the implementation of new methodological approaches during the COVID-19 outbreak, as face-to-face visits were not possible. One key question for AD researchers looking to implement remote trials was whether this change would be feasible with an older adult population. There are stereotypes associated with older adults and technology use, which are often negative and framed around their supposed inability to adapt to and adopt new forms [20]. Despite barriers which may prevent older adults from using new digital technologies, the implementation of remote assessments over the COVID-19 lockdown period was successful within the CHARIOT PRO Substudy (CPRO SS [21]). Therefore, this study aimed to explore differences in technology readiness of participants enrolled in the CPRO SS who completed virtual visits during the lockdown period. These results could inform future AD prevention studies that aim to implement similar remote technologies.

## Methods

### Study design

The CPRO SS is a longitudinal, observational, and AD biomarker-enriched study of cognitively unimpaired older adults at risk of AD in which participants are invited in person to administer samples, complete cognitive testing, and complete self-report measures [21]. On the 23rd of March 2020, the UK initiated its first lockdown in an attempt to mitigate the spread of COVID-19 (“*Timeline of UK government coronavirus lockdowns and restrictions*”; Institute for Government). Following this, the CPRO SS transitioned to a remote protocol by following the legal and scientific guidance at the time [21]. A significant protocol adaptation allowed for the maintenance of operational activities, including cognitive assessments, clinical evaluations, and self-reported lifestyle questionnaires (querying diet, physical activity, sleep, mood, and activities of daily living [21]). In facilitating the shift from study site visits to remote online visits, a crucial consideration was the technical capacity and capabilities of participants. To measure various aspects of technology-related behaviour, the CHARIOT Technology Questionnaire (CTQ) was developed to assess participants’ attitudes, feelings, and experiences of technology, including online teleconference platforms (e.g., Microsoft Teams™, Zoom™, and Skype™).

### Study participants

Male and female older adults aged 60 and over were invited to complete the CTQ. Active participants (*n* = 326) from the CPRO SS, as of March 2020, were based in Greater London and South-West England.

### Study outcome measures

#### CHARIOT Technology Questionnaire (CTQ)

The CTQ was developed primarily as a voluntary self-report tool to understand participants’ attitudes and experiences using technology. It was considered a supplementary outcome measure outside of the typical CPRO SS protocol. The CTQ was distributed to participants who had completed at least one “full” visit before the time of CTQ dissemination. This was defined as a participant completing a remote session comprising at least one cognitive assessment, followed by the completion of online self-report lifestyle questionnaires.

Thematically, CTQ covered four areas across three sections: demographics, prior technology use, current technology use, and reflections on the CPRO SS remote protocol. Section 1a. comprised of demographic questions (gender and age). Section 1b. included questions on technology use (including online teleconference platforms and typical weekly internet use). Section 2. included questions specifically about their thoughts and feelings towards the CPRO SS remote sessions (defined as a session comprising of at least one remote cognitive assessment and online questionnaire). Section 3. covered questions on self-report outcomes (e.g., lifestyle questionnaires). CTQ contained a maximum of 51 questions (depending on participants’ responses). CTQ also adapted nine questions from the Technology Readiness Index (TRI; [22], *see *Table 1*.*) as Likert scales with options ranging from “Strongly Disagree” to “Strongly Agree” (e.g., “In general, I am among the first in my circle of friends to acquire new technology” [“Strongly Disagree” = 1, “Neutral” = 3, and “Strongly Agree” = 5]).

**Table 1:** Technology readiness index

	**Strongly Disagree**	**Somewhat Disagree**	**Neutral**	**Somewhat Agree**	**Strongly Agree**
a. In general, I am among the first in my circle of friends to acquire new technology	1	2	3	4	5
b. I can usually figure out new “high-tech” products and services without help from others	1	2	3	4	5
c. I find new technologies to be mentally stimulating	1	2	3	4	5
d. If I provide information to a machine or over the internet, I can never be sure it really gets to the right place	1	2	3	4	5
e. I like computer programmes that allow me to tailor things to fit my own needs	1	2	3	4	5
f. Other people come to me for advice on new technologies	1	2	3	4	5
g. I do not consider it safe to do any kind of financial business online	1	2	3	4	5
h. I worry that information I send over the internet will be seen by other people	1	2	3	4	5
i. It is embarrassing when I have trouble with a “high-tech” gadget while people are watching	1	2	3	4	5

A nine-item version of the TRI was selected over longer alternatives to minimise participant burden. This shortened form retains the core dimensions of the original instrument while enhancing practicality, especially when administered alongside other CTQ items. It was judged to strike an appropriate balance between comprehensiveness and accessibility, encouraging accurate completion without causing fatigue or disengagement.

## Results

### Demographics

A total of 226 out of 326 participants took part in this voluntary component of the CPRO SS (overall response rate = 69.33%). For the purposes of the current analyses, the analytic sample includes the 226 participants who consented to and completed the CTQ. The remaining 100 participants did not participate in this remote component due to a lack of consent.

The gender split was 100 (44.25%) and 126 (55.75%) females and males, respectively. Participants’ age ranged from 63 to 89 (*M* = 75.60 years, *SD* = 5.33 years). Participants were mostly White British (*n* = 210).

### Technology readiness

All analyses were conducted in Jamovi (version 2.5.6) [23].

The internal reliability of the TRI questionnaire was assessed using Cronbach’s alpha. The overall internal reliability was acceptable, with a total Cronbach’s alpha of *α* = 0.81. Reliability was also acceptable across all questions: TRI A (*α* = 0.79), TRI B (*α* = 0.76), TRI C (*α* = 0.78), TRI D (*α* = 0.79), TRI E (*α* = 0.80), TRI F (*α* = 0.77), TRI G (*α* = 0.79), TRI H (*α* = 0.81), and TRI I (*α* = 0.82).

**Fig. 1:**
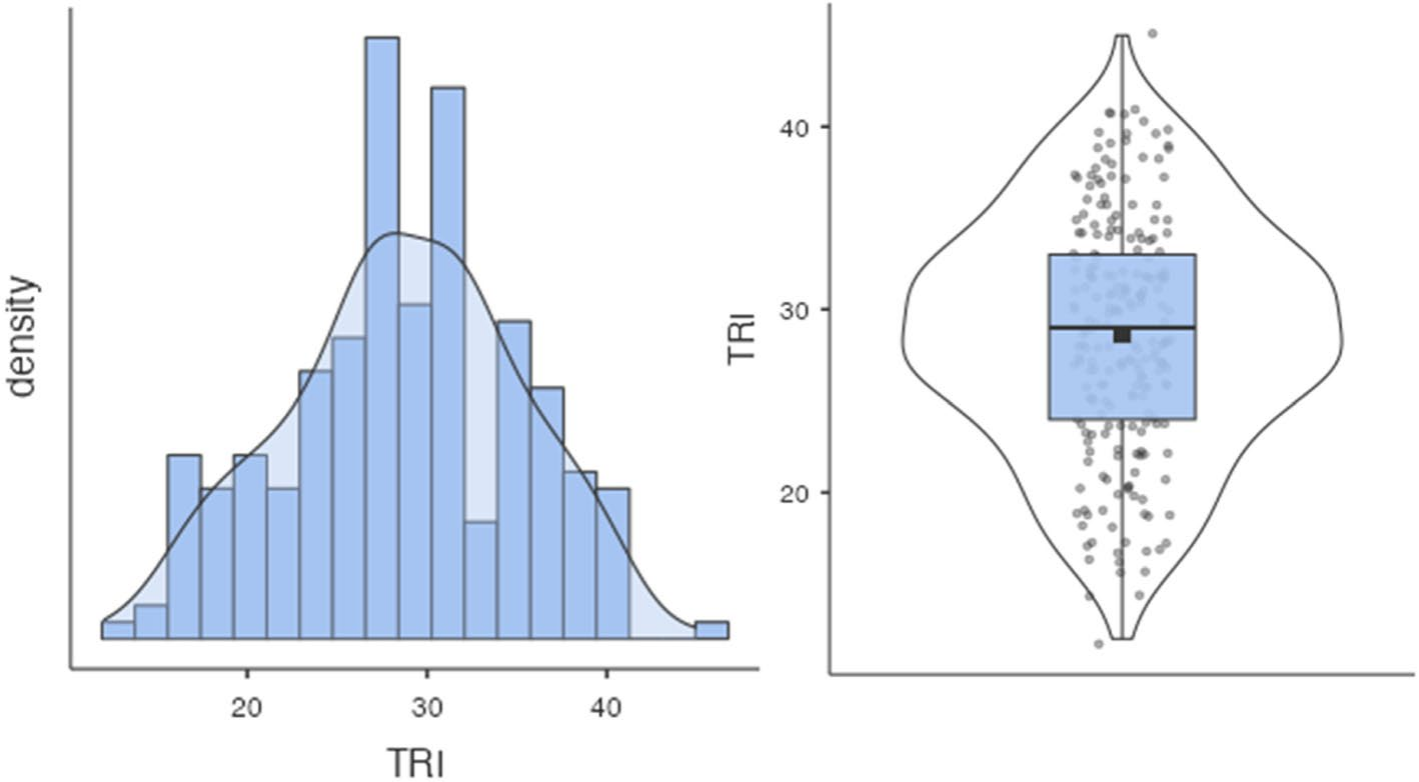
Distribution of Technology Readiness Index (TRI) scores

To create a composite score for the TRI, scores were summed for each item, ensuring that negatively worded items (e.g., D, G, H, and I) were reverse-coded so higher scores consistently reflected greater technology readiness. The mean TRI score was 28.60 (*SD* = 6.48) (*see also *Fig. 1). Total TRI differed between genders, as on average, female participants scored lower technology readiness (*M* = 27.50, *SD* = 6.87) compared to males (*M* = 29.50, *SD* = 6.02).

An independent *t*-test was used to determine whether there was any significant difference in TRI scores between males and females. Results showed that females had significantly lower TRI scores compared to males, *t*(224) = −2.30, *SE* = 0.859, *p* = 0.022. The effect size was small to moderate, *d* = −0.31. Next, simple linear regression found that age significantly predicted TRI,* β* = −0.199, *t* = −2.48, *p* = 0.014. The effect size was small, with *R*^*2*^ = 0.03.

Exploratory factor analysis (EFA) was then used to explore the factor loadings of TRI A to I with a direct oblimin rotation (Table 2). The scree plot showed a steep fall in the amount of variance explained past factor two (Factor 1 = 3.121, Factor 2 = 0.775, Factor 3 = 0.102… Factor 9 = −0.407). We, therefore, opted to use two factors for subsequent analyses. Factor 1 was labelled “Technology Competence"and was comprised of TRI sub-scales A to C and E to F, and Factor 2 was labelled “Technology Trepidation” and was comprised of TRI sub-scales D and G to I. “Technology Competence” and “Technology Trepidation” scores were then *z*-transformed for each participant by using the overall sample mean and *SD* for each respective factor. *Z*-scores were then used to investigate whether differences in “Technology Competence” and “Technology Trepidation” were related to gender and age. Correlations between individual TRI items, assessed using Spearman's rank due to non-normal distribution, are presented in Table 3.

**Table 2:** Factor loadings for technology readiness index questions A-I

	**Factor**		
	**1**	**2**	**Uniqueness**
*TRI A*	.662		.556
*TRI B*	.831		.282
*TRI C*	.702		.491
*TRI D*		.433	.623
*TRI E*	.607		.661
*TRI F*	.801		.381
*TRI G*		.485	.602
*TRI H*		.867	.277
*TRI I*		.396	.844

*TRI* Technology Readiness Index

‘Minimum residual’ extraction method was used in combination with an'oblimin'rotation

**Table 3:** Correlation matrix between technology readiness index sub-scales

		**TRI A**	**TRI B**	**TRI C**	**TRI D**	**TRI E**	**TRI F**	**TRI G**	**TRI H**	**TRI I**
*TRI A*	Spearman's rho	-								
	*p*-value									
*TRI B*	Spearman's rho	.572	-							
	*p*-value	<.001								
*TRI C*	Spearman's rho	.467	.568	-						
	*p*-value	<.001	<.001							
*TRI D*	Spearman’s rho	-.303	-.359	-.363	-					
	*p*-value	<.001	<.001	<.001						
*TRI E*	Spearman's rho	.331	.486	.393	-.203	-				
	*p*-value	<.001	<.001	<.001	<.001					
*TRI F*	Spearman’s rho	.568	.690	.524	-.324	.431	-			
	*p*-value	<.001	<.001	<.001	<.001	<.001				
*TRI G*	Spearman's rho	-.222	-.410	-.336	.463	-.227	-.323	-		
	*p*-value	<.001	<.001	<.001	<.001	<.001	<.001			
*TRI H*	Spearman's rho	-.196	-.229	-.171	.431	.020	-.133	.489	-	
	*p*-value	<.001	<.001	<.001	<.001	.592	<.001	<.001		
*TRI I*	Spearman's rho	-.044	-.112	-.080	.216	.013	-.127	.193	.351	-
	*p*-value	.243	.003	.034	<.001	.723	<.001	<.001	<.001	

*TRI* Technology Readiness Index, TRI001 to TRI009 = TRI questions 1 to 9

The results for Technology Competence revealed a significant gender difference. An independent samples *t*-test showed that females (*M* = −0.205, *SD* = 0.990) scored significantly lower than males (*M* = 0.153, *SD* = 0.973), *t*(224) = −2.75, *p* = 0.006, and the effect size was small to moderate, *d* = −0.37. Because the Technology Trepidation scores were non-normally distributed, as indicated by Shapiro–Wilk tests, a non-parametric Mann–Whitney U test was conducted to compare the scores between males and females. The test revealed no significant difference in Technology Trepidation scores between males and females, *p* = 0.474. The effect size was negligible, *r*_*s*_ = 0.05.

A simple linear regression was used to determine whether age predicted “Technology Competence”. The analysis did not reveal a significant relationship, with age not emerging as a statistically significant predictor, *β* = −0.022, *t* = −1.81, *p* = 0.072. The effect size was small, *R*^*2*^ = 0.01. Furthermore, a robust linear regression was conducted to examine the relationship between age and Technology Trepidation. The analysis revealed that age was a statistically significant predictor of Technology Trepidation, *β* = −0.173, *t* = −2.57, *p* = 0.011, although the effect size was small (*R*^*2*^ = 0.03).

In summary, EFA supported a two-factor structure of the TRI: “Technology Competence” and “Technology Trepidation.” Gender was significantly associated with Technology Competence, with females scoring lower than males. No significant gender differences were found for Technology Trepidation. Age did not significantly predict Technology Competence but was significantly associated with lower Technology Trepidation. These findings highlight the influence of gender and age on specific aspects of technology readiness in older adults.

## Discussion

This study aimed to (a) assess technology readiness among a sample of cognitively unaffected older adults participating in an AD prevention observational trial and (b) determine whether there were differences in technology readiness based on distinct demographic factors (specifically gender and age). Our results suggest that both gender and age are associated with differences in technology readiness. Specifically, female participants had, on average, lower TRI scores, and age was a significant predictor of technology readiness. Because of our White ethnocentric sample, potential differences in ethnicity categories and TRI scores could not be determined.

While the effect sizes associated with these demographic variables were modest, they revealed consistent and meaningful patterns that enhance our understanding of the factors influencing technology-related attitudes and behaviours. For example, gender differences in TRI scores (*d* = −0.31) and Technology Competence (*d* = −0.37) fall within the small to moderate range, suggesting that gender may play a role in shaping these perceptions. Similarly, age explained a small but statistically significant portion of the variance in TRI scores (*R*^*2*^ = 0.03), suggesting it is a relevant factor within a broader, more complex set of influences. These findings offer a valuable stepping-stone for future research, which can expand upon this foundation by exploring additional variables and leveraging larger, more diverse samples to deepen insights and inform practical applications.

TRI sub-scales loaded onto two factors, and we defined these as “Technology Competence” and “Technology Trepidation”. These factors described technology understanding and negative affective responses to technology, respectively. “Technology Competence” and “Technology Trepidation” may inform future strategies when supporting older adults in adopting technology for remote protocols in AD research. By increasing “Technology Competence” and decreasing “Technology Trepidation”, we predict that older adults would feel more confident in adopting technology for the purposes of participation in remote AD research. Increased technology readiness may have downstream benefits for clinical healthcare systems in delivering health outcomes for older adults, with telemedicine being described as a significant factor in reducing future NHS spending [24, 25].

Gender-split analyses found that female participants, on average, scored lower on technology readiness, suggesting a gender-based imbalance in technology readiness. Women have a higher prevalence of AD than men [26], meaning it is pertinent to address gender differences in technology readiness. This has potential wider applications to healthcare, as telemedicine and assessment approaches have shown a rise in popularity [27]. A trend catalysed by the COVID-19 pandemic [28]. However, gender is not a homogenous group [29]. Further research with intersectionality-led approaches is needed to isolate smaller sub-samples in need of support and to consider gender heterogeneity, with sub-groups potentially having different technology-based needs [29]. Furthermore, previous research found that women with a mean age of 34 (categorised as ‘younger adults’) showed higher e-literacy and health telemedicine satisfaction compared to men [30]. This may suggest that levels of technology readiness are not consistent over one’s lifetime. This sample was derived from Southern Israel and may also point towards cultural differences in constructs surrounding the readiness to adopt technology-based approaches and/or interactions between gender, age, and culture.

Furthermore, we found that "Technology Competence" differences may be an underlying mechanism behind lower TRI scores among female participants. Ranieri et al. [31] postulated the link between technology use among older adults and cognitive reserve. Cognitive reserve is defined as the extent to which cognition is preserved despite the presence of neural damage [32]. Higher education, occupational complexity, and greater engagement with stimulating activities are associated with higher cognitive reserve [33]. Higher cognitive reserve could be linked to greater technology use in daily life and more efficient neural processing (thus preserving cognition among older adults for longer [31]. Previous generations of women had less access to cognitive reserve contributors, such as higher levels of education or roles with high occupational complexity [34]. Despite this, some studies have reported that women have higher cognitive reserve [35]. Whether differences in cognitive reserve are associated with technology readiness is yet to be determined.

There is an ageing population in many societies [36], and our current study found age as a significant predictor of technology readiness. Interestingly, there were also age-related effects pertaining to “Technology Trepidation” (Factor 1) but not “Technology Competence” (Factor 2). A barrier to technology readiness may be the negative feelings associated with technology experienced by older adults with advancing age. Mariano et al. [20] found “stereotype threat” as a significant factor affecting technology use among older adults. This was negatively associated with perceived ease of technology use, to which anxiety mediated this relationship. This suggests that lower technology use among older adults may be due to fear of confirming negative stereotypes, and these social factors may be a significant barrier to technology use in later life [20]. Self-esteem, relationships, and political scepticism are other variables that could impact technology use among older adults [37]. Conversely, evidence suggests that digitalisation, necessitated by the COVID-19 pandemic, may have helped to change older adults’ perceptions of digital technology for the better [38], including broadening social relationships [39]. Hence, there is an argument in favour of improving older adults’ technological abilities for personal well-being, alongside the beneficial effects of cognitive training and engagement, as well as social connectivity.

The emerging trend for virtual visits in clinical research may lead to a cheaper and more streamlined approach to recruiting participants for trials. A significant driver of this trend was catalysed by the COVID-19 pandemic (“*The impact of Covid-19 on the use of digital technology in the NHS*”, [40]). For example, the E-FINGER PRINT study is a five-year large-scale virtual feasibility study with potential benefits of economic sustainability and up-scalability via the use of remote assessments [41]. The opportunities for adopting remote assessments (e.g., via accessible technologies) are widespread, as exemplified by the CAN-THUMBS UP (a Canadian branch of the World-Wide Fingers trial), which is adopting the use of videoconferencing technology, remote cognitive testing, wearable devices, and self-administered saliva sample collection methods [42]. Remote assessments can ultimately recruit across wider geographical areas and more diverse populations [43].

This study is not without limitations. Our study sample mostly consisted of White participants (particularly White British). Therefore, we cannot attempt to extrapolate the study results to the general population comprising a diverse range of ethnic groups. Future studies may wish to replicate our research with more ethnically diverse samples to determine ethnically specific group differences in technology readiness. This could potentially identify social inequities in technology readiness and strategies for how these can be overcome. Other demographic factors, including socioeconomic status and education levels, should also be considered.

Furthermore, there may have been participants who experienced technical issues with hardware, internet access, etc., and who may have struggled to complete the CTQ over the period of data collection. However, we expect the proportion of these participants to be small (if at all present), as all active participants (who, therefore, were invited to complete the CTQ) were in correspondence with CPRO SS staff for other research-based activities over the period of CTQ data collection.

## Conclusions

Remote assessments in clinical research and telemedicine-based approaches in clinical healthcare systems are gaining increasing popularity. The CPRO SS, AD prevention trial, utilised remote assessments throughout the COVID-19 pandemic. Participants’ technology readiness levels were found to differ by gender and age. EFA grouped various TRI sub-scales into two factors, and these factors showed different relationships based on participants’ gender and age.

Future AD trials implementing remote protocols, such as remote neuropsychological testing, may benefit in measuring technology readiness. This may improve overall participant inclusion and reduce attrition rates among those with lower levels of technology readiness. Providing support may increase feelings of technology competence, reduce technology trepidation, and facilitate reliable and valid measures of participants’ cognitive performance during remote protocols. Overall, supporting participants in adopting the necessary technology and adapting to its use may improve their access to electronic services. This may prove instrumental in utilising remote assessments in AD prevention trials moving forward.

## Data Availability

The datasets generated and analysed during the current study are not publicly available due to the data being privately funded, and explicit permission for public access and use has not been granted at this time. However, they are available from the corresponding author upon reasonable request.

## Abbreviations

AD: Alzheimer’s Disease
COVID-19: Coronavirus disease 2019
CPRO SS: CHARIOT PRO Substudy
CTQ: CHARIOT Technology Questionnaire
EFA: Exploratory Factor Analysis
NHS: National Health Service
TRI: Technology Readiness Index
UK: United Kingdom
WHO: World Health Organisation
